# Relationship between Early Physician Follow-Up and 30-Day Readmission after Acute Myocardial Infarction and Heart Failure

**DOI:** 10.1371/journal.pone.0170061

**Published:** 2017-01-27

**Authors:** Yu-Chi Tung, Guann-Ming Chang, Hsien-Yen Chang, Tsung-Hsien Yu

**Affiliations:** 1 Institute of Health Policy and Management, National Taiwan University, Taipei, Taiwan; 2 Department of Family Medicine, Cardinal Tien Hospital, New Taipei City, Taiwan; 3 School of Medicine, Fu Jen Catholic University, New Taipei City, Taiwan; 4 Department of Health Policy and Management, Center for Drug Safety and Effectiveness, Johns Hopkins Bloomberg School of Public Health, Baltimore, United States of America; 5 Department of Health Care Management, National Taipei University of Nursing and Health Sciences, Taipei, Taiwan; University of Messina, ITALY

## Abstract

**Background:**

Thirty-day readmission rates after acute myocardial infarction (AMI) and heart failure are important patient outcome metrics. Early post-discharge physician follow-up has been promoted as a method of reducing 30-day readmission rates. However, the relationship**s** between early post-discharge follow-up and 30-day readmission for AMI and heart failure are inconclusive. We used nationwide population-based data to examine associations between 7-day physician follow-up and 30-day readmission, and further associations of 7-day same physician (during the index hospitalization and at follow-up) and cardiologist follow-up with 30-day readmission for non-ST-segment-elevation myocardial infarction (NSTEMI) or heart failure.

**Methods:**

We analyzed all patients 18 years or older with NSTEMI and heart failure and discharged from hospitals in 2010 in Taiwan through Taiwan’s National Health Insurance Research Database. Cox proportional hazard models with robust sandwich variance estimates and propensity score weighting were performed after adjustment for patient and hospital characteristics to test associations between 7-day physician follow-up and 30-day readmission.

**Results:**

The study population for NSTEMI and heart failure included 5,008 and 13,577 patients, respectively. Early physician follow-up was associated with a lower hazard ratio of readmission compared with no early physician follow-up for patients with NSTEMI (hazard ratio [HR], 0.47; 95% confidence interval [CI], 0.39–0.57), and for patients with heart failure (HR, 0.54; 95% CI, 0.48–0.60). Same physician follow-up was associated with a reduced hazard ratio of readmission compared with different physician follow-up for patients with NSTEMI (HR, 0.56; 95% CI, 0.48–0.65), and for patients with heart failure (HR, 0.69; 95% CI, 0.62–0.76).

**Conclusions:**

For each condition, patients who have an outpatient visit with a physician within 7 days of discharge have a lower risk of 30-day readmission. Moreover, patients who have an outpatient visit with the same physician within 7 days of discharge have a much lower risk of 30-day readmission.

## Introduction

Policy makers, clinicians, and payers who seek to improve outcomes in health care are focusing on 30-day readmission rates for patients with acute myocardial infarction and those with heart failure.[1] Early post-discharge physician follow-up has been promoted as a method of reducing readmission rates.[2] However, studies on the relationships between early post-discharge follow-up and patient outcomes for acute myocardial infarction and heart failure are not only rare but also inconclusive. Of only two studies we are aware of on this topic, one showed that discharge from hospitals that have higher early follow-up rates is associated with a reduction in 30-day readmission for heart failure,[3] but another did not establish such relationship for acute myocardial infarction.[4] Moreover, to our knowledge, no study has examined whether early follow-up with the same physician or with a cardiologist is associated with lower 30-day readmission for patients with acute myocardial infarction and those with heart failure.

Early or timely outpatient follow-up with a physician for further assessment or treatment has been hypothesized to have an effect on reduced readmission among discharged patients with heart failure and those with acute myocardial infarction.[3–7] The transition from hospital to home is a period of particularly higher risk. Timely post-discharge follow-up has been suggested as an important component of efforts to optimize transitional care during the high-risk peri-discharge period.[8, 9] With physiological stress and allostatic load derived from hospitalization, the risks in the critical 30-day period after discharge might exist.[10] More importantly, the immediate days that follow discharge are also a vulnerable period owing to the additional therapies or changes in existing medical therapy that may worsen patient outcomes.[3, 11] Seven-day follow-up with the physicians may have the benefit of improving patient outcomes through providing clinical interventions on disease instability (such as diagnostic testing and medication changes),[12] and 7-day follow-up with the same physician (because of physician continuity) or with a specialist (because of physician specialty) may be associated with better outcomes than follow-up with other physicians.[3, 4] However, few studies have examined the relationship between 7-day physician follow-up and patient outcomes.

In Taiwan, the National Health Insurance Administration has been the sole insurer and implemented national health insurance since March 1, 1995. The coverage rate of national health insurance has reached 99.9%, and almost all health care facilities are national health insurance contracted providers. Every enrollee is free to go to any hospital or clinic, and enjoys comprehensive benefits with a low cost-sharing policy (10% coinsurance for inpatient care with a yearly cap of about US$1700, and a US$1.7–12.0 copayment for outpatient visits). The National Health Insurance Administration has reimbursed providers mainly on a fee-for-service basis since the beginning of the national health insurance program. The Hospital Readmissions Reduction Program, which, under the Affordable Care Act, requires the Centers for Medicare & Medicaid Services to reduce payments to hospitals with excessive readmission rates regarding acute myocardial infarction and heart failure,[13] has not yet been introduced in Taiwan. Therefore, Taiwan’s healthcare system provides an excellent opportunity to examine whether early post-discharge follow-up improves patient outcomes for patients with acute myocardial infarction and those with heart failure.

To better understand the relationship between early post-discharge follow-up and patient outcomes, we used nationwide population-based data from Taiwan to examine whether physician follow-up within 7 days of discharge was associated with a reduction in 30-day readmission for patients with acute myocardial infarction and those with heart failure. We also further determined whether early follow-up with the same physician or with a cardiologist was associated with much better patient outcomes.

## Methods

### Database

In this study, we used the National Health Insurance Research Database, provided by the National Health Insurance Administration and managed by the National Health Research Institutes in Taiwan. The database, which is released annually, consists of the following dimensions: inpatient medical benefit claims, ambulatory care medical benefit claims, pharmaceutical benefit claims, contracted medical care institutions, health professionals in contracted medical care institutions, and beneficiaries. Therefore, the database provides an opportunity to examine the relationship between early post-discharge follow-up and patient outcomes.

### Ethical Statement

The protocol for this study was approved by the Institutional Review Board of the National Taiwan University Hospital (protocol # 201601056RINB). The dataset we used in this study was secondary data; all information was de-identified by data owners.

### Study Population

This study included all patients aged 18 years and over who were discharged from general acute care hospitals in 2010, with a principal diagnosis of acute myocardial infarction without ST-segment elevation (International Classification of Diseases, 9th revision, clinical modification [ICD-9-CM] code 410.7)[4, 14–16] and heart failure with or without preserved ejection fraction (ICD-9-CM code 428), respectively.[17–20] Only the first admission of patients with multiple hospitalizations was included for the same medical condition.[3, 4] We excluded patients who were admitted with the same medical condition during the past 6 years, died in the hospital, were transferred out, left the hospital against medical advice, or were discharged against medical advice in a terminally critical condition.[4, 21]

### Measures of Variables

#### Early follow-up

Early follow-up was defined as whether discharged patients had an outpatient visit with a physician within 7 days after discharge.[3, 4] The length of seven days was selected to be consistent with current efforts to improve transitional care.[22] Early follow-up with the same physician was measured as whether discharged patients visited the same physician during the hospitalization and during early follow-up.[3] Patients who visit the same physician during the hospitalization and at follow-up are considered to have better continuity of care.[3, 19] Early follow-up with a cardiologist was defined as whether discharged patients visited a cardiologist within 7 days after discharge.[3, 4] To explore whether the combined effect of same physician follow-up and cardiologist follow-up on patient outcomes, the interaction term between same physician follow-up and cardiologist follow-up was also considered.

#### Patient and hospital characteristics

The covariates included patient and hospital characteristics. The patient covariates included sex, age, comorbid conditions, medical history, in-hospital treatment (percutaneous coronary intervention use, intensive care unit use, administration of surgical operation), length of stay, baseline medications (aspirin, β-blocker, statin, angiotensin-converting enzyme inhibitor/angiotensin receptor blocker), medications within 7 days of discharge, low income, rural residence, number of hospitalizations during the past year, and number of office visits during the past year.[3, 4, 9, 19, 23–26].

The Charlson-Deyo index was used to quantify patient comorbidities.[27] This index is the sum of the weighted scores based on the presence or absence of 17 different medical conditions during the past year and the index hospitalization. A score of 0 means that no comorbid index is present, and higher scores point to a greater burden of comorbidity. In addition, we also measured patients’ medical history specially related to cardiovascular conditions during the past year and the index hospitalization, which included cardiac risk factors (hypertension, diabetes mellitus), prior cardiac conditions (myocardial infarction, heart failure, atrial fibrillation or flutter), and medical comorbidities (peripheral vascular disease, renal disease).[28] The use of the baseline medications was measured according to whether the medications were prescribed during hospital stays and at discharge. Low income was measured as whether the patient was enrolled as a low-income beneficiary. Rural residence was measured whether the patient lived in a rural area based on previous studies using National Health Insurance Research Database.[29–32]

The hospital covariates included hospital volume (low, medium, high), hospital level (academic medical center, regional, district), teaching status (yes/no), and geographic location (Taipei, northern, central, southern, Kao-Ping, eastern). We determined the volume of patients who were treated at hospitals using annual condition-specific volume, and then we divided these “annualized” volumes into tertiles.

#### Outcome measures

Our primary outcome was 30-day all-cause readmission, which was defined as any re-hospitalization to any acute care hospital within 30 days from index discharge.[3, 4] Hospital readmissions are regarded as potential indicators of poor care or missed opportunities to better coordinate care.[33, 34] Additionally, readmission is expensive to the health care system and commonly represents a preventable adverse event for patients.[24] Secondary outcomes of interest were readmission within 30 days of discharge for cardiovascular cause (primary and secondary ICD-9-CM codes 390–459)[17, 35] and readmission within 30 days of discharge for the same cause as the index hospitalization.

### Statistical Analysis

We used Cox proportional hazard models with robust sandwich variance estimates and propensity score weighting, adjusted for all patient and hospital characteristics, to examine the association between 7-day physician follow-up and 30-day readmission for acute myocardial infarction and heart failure.[28, 36] Cox proportional hazard models allows us to take into account the length of survival after discharge to avoid the problem that patients with early visit are the most severe ones and then they were less likely to be re-hospitalized because they die more often. The models focused on time from discharge until the first re-hospitalization date during the 30 days of follow-up. Patients were censored on date of death, or 30 days post-discharge, whichever came first. All analyses were adjusted for clustering at the hospital level with the use of robust sandwich variance estimates. We modeled 30-day readmission as a function of 7-day physician follow-up, sex, age, comorbid conditions, medical history, in-hospital treatment, length of stay, baseline medications, low income, rural residence, number of hospitalizations during the past year, number of office visits during the past year, hospital volume, hospital level, teaching status, and geographic location.

Moreover, we used propensity score analyses to reduce the selection bias and the potential baseline differences between the early follow-up and the no early follow-up groups.[9, 28] Propensity scores were computed by modeling a logistic regression model in which the dependent variable was whether the discharged patient had an outpatient visit with a physician within 7 days after discharge. The independent variables were patient sex, age, comorbid conditions, medical history, in-hospital treatment, length of stay, baseline medications, low income, rural residence, number of hospitalizations during the past year, number of office visits during the past year, hospital volume, hospital level, teaching status, and geographic location. Then, each patient was weighted by the inverse propensity score when performing Cox proportional hazard models with robust sandwich variance estimates to reduce the selection bias.[25, 37–40]

Finally, we further used Cox proportional hazard models with robust sandwich variance estimates, adjusted for all patient (including medications after discharge) and hospital characteristics, to examine the individual and combined associations of early same physician and early cardiologist follow-up with 30-day readmission among the early follow-up group. In sensitivity analyses, we changed the definition of early physician follow-up by allowing early follow-up to occur within 14 days after discharge.[3] Statistical significance was indicated by a 2-tailed value of P<0.05. All analyses were performed with SAS version 9.3 (SAS Institute Inc, Cary, NC).

## Results

### Baseline Characteristics and Outcomes

**Fig 1:**
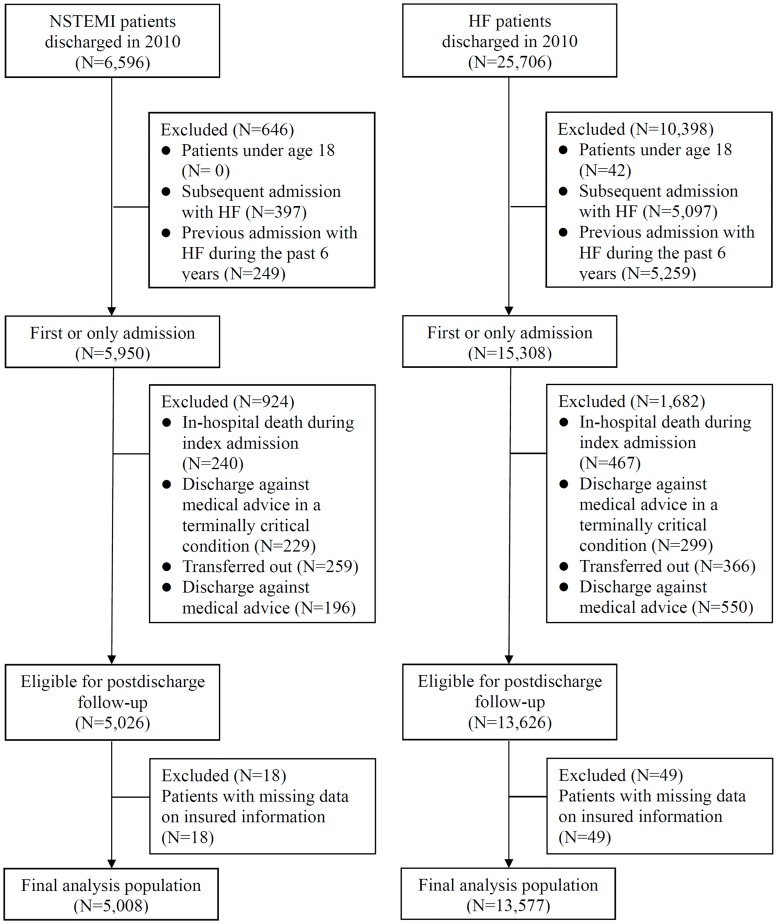
Flow diagram of patient selection. Shown are inclusions and exclusions and the final study cohort. HF indicates heart failure; NSTEMI, non-ST-segment-elevation myocardial infarction.

Regarding non-ST-segment-elevation myocardial infarction (NSTEMI), we identified 6596 patients discharged from hospital in 2010. We excluded patients under age 18 (N = 0), and patients who had subsequent admission with NSTEMI (N = 397), or had previous admission with NSTEMI during the past 6 years (N = 249). After exclusions (Fig 1), the final NSTEMI population included 5008 patients. The final population with heart failure consisted of 13577 patients.

Table 1 presents these patient characteristics. Among patients with NSTEMI, rates of early follow-up with a physician, with the same physician, and with a cardiologist were 76.7%, 56.1%, and 44.8%, respectively. Among those with heart failure, rates of early follow-up with a physician, with the same physician, and with a cardiologist were 74.9%, 52.9%, and 37.6%, respectively. Thirty-day all-cause readmission rates for patients with NSTEMI and those with heart failure were 19.9% and 23.3%, respectively.

**Table 1:** Characteristics of the study population.

	NSTEMI	Heart failure
	N(%)	N(%)
No. of patients	5008(100.0)	13577(100.0)
Follow-up with a physician		
7-day	3841(76.7)	10164(74.9)
14-day	4664(93.1)	12197(89.8)
Follow-up with the same physician		
7-day	2807(56.1)	7178(52.9)
14-day	3614(72.2)	8996(66.3)
Follow-up with a cardiologist		
7-day	2242(44.8)	5103(37.6)
14-day	2862(57.1)	6455(47.5)
Patient Characteristics		
Male sex	3367(67.2)	6824(50.3)
Age, y		
18–49	520(10.4)	1026(7.6)
50–64	1456(29.1)	2345(17.3)
65–79	1920(38.3)	5058(37.3)
80+	1112(22.2)	5148(37.9)
Charlson score		
0–1	2181(43.6)	3838(28.3)
2–3	1518(30.3)	4988(36.7)
4+	1309(26.1)	4751(35.0)
Medical history		
Hypertension	3829(76.5)	10617(78.2)
No	1179(23.5)	2960(21.8)
Diabetes mellitus	2439(48.7)	6194(45.6)
No	2569(51.3)	7383(54.4)
Myocardial infarction	650(13.0)	1304(9.6)
No	4358(87.0)	12273(90.4)
Heart Failure	827(16.5)	5930(43.7)
No	4181(83.5)	7647(56.3)
Atrial fibrillation or flutter	511(10.2)	3889(28.6)
No	4497(89.8)	9688(71.4)
Peripheral vascular disease	182(3.6)	564(4.2)
No	4826(96.4)	13013(95.8)
Renal disease	923(18.4)	2697(19.9)
No	4085(81.6)	10880(80.1)
In-hospital treatment		
PCI	2893(57.8)	458(3.4)
Intensive care unit use	4311(86.1)	2969(21.9)
Surgery use	471(9.4)	582(4.3)
Length of stay, d		
<5	2039(40.7)	5671(41.8)
6–9	1512(30.2)	4366(32.2)
10+	1457(29.1)	3540(26.1)
Baseline medications		
Aspirin	4710(94.0)	6573(48.4)
β-Blocker	3183(63.6)	5268(38.8)
Statin	2961(59.1)	2283(16.8)
ACEI/ARB	3610(72.1)	7995(58.9)
Medications within 7 days of discharge		
Aspirin	2590(51.7)	3571(26.3)
β-Blocker	1832(36.6)	3233(23.8)
Statin	1670(33.3)	1292(9.5)
ACEI/ARB	1792(35.8)	4305(31.7)
Medications within 14 days of discharge		
Aspirin	3425(68.4)	4637(34.2)
β-Blocker	2450(48.9)	4380(32.3)
Statin	2272(45.4)	1752(12.9)
ACEI/ARB	2403(48.0)	5660(41.7)
Low income	98(2.0)	305(2.2)
Rural residence	1205(24.1)	3687(27.2)
Number of hospitalizations during the past year		
0	3312(66.1)	6935(51.1)
1	942(18.8)	3344(24.6)
2+	754(15.1)	3298(24.3)
Number of outpatient visits during the past year		
Low	1696(33.9)	4610(34.0)
Medium	1648(32.9)	4447(32.8)
High	1664(33.2)	4520(33.3)
Hospital Characteristics		
Hospital volume		
Low	1685(33.6)	4675(34.4)
Medium	1693(33.8)	4575(33.7)
High	1630(32.5)	4327(31.9)
Hospital level		
Academic medical center	2797(55.9)	4571(33.7)
Regional	2077(41.5)	6984(51.4)
District	134(2.7)	2022(14.9)
Teaching	4939(98.6)	12003(88.4)
Location		
Taipei	1743(34.8)	4608(33.9)
Northern	581(11.6)	1841(13.6)
Central	799(16.0)	2534(18.7)
Southern	858(17.1)	2054(15.1)
Kao-Ping	855(17.1)	2073(15.3)
Eastern	172(3.4)	467(3.4)
Patient outcomes		
30-day readmission		
All-cause	998(19.9)	3169(23.3)
Specific-cause		
Cardiovascular	935(18.7)	2842(20.9)
Same diagnosis	182(3.6)	1983(14.6)

NSTEMI indicates non-ST-segment-elevation myocardial infarction; and PCI, percutaneous coronary intervention.

### Associations of Early Follow-Up with Patient and Hospital Characteristics

Table 2 shows the comparison of the discharges followed by outpatient visits with a physician within 7 days to those without. For NSTEMI, baseline characteristics that differed between the follow-up and the no follow-up groups were medical history (heart failure, atrial fibrillation or flutter),PCI use, length of saty, statin use, number of outpatient visits during the past year, hospital volume, level, and location. For heart failure, baseline characteristics that differed between the follow-up and the no follow-up groups were medical history (hypertension, heart failure), PCI use, length of stay, number of hospitalizations during the past year, number of outpatient visits during the past year, hospital volume, level, teaching status, and location. After propensity score weighting, the two groups for NSTEMI and heart failure were well balanced for the baseline characteristics.

**Table 2:** Baseline characteristics by early follow-up.

	NSTEMI						Heart failure					
	Unweighting			Weighting^a^			Unweighting			Weighting^a^		
	Follow-up			Follow-up			Follow-up			Follow-up		
	Yes, %	No, %	P value	Yes, %	No, %	P value	Yes, %	No, %	P value	Yes, %	No, %	P value
Patient Characteristics												
Sex												
Male	67.1	67.7	0.701	67.2	66.7	0.602	50.0	51.0	0.312	50.2	49.8	0.495
Female	32.9	32.3		32.9	33.3		50.0	49.0		49.8	50.2	
Age, y												
18–49	10.3	10.8	0.642	10.4	10.9	0.851	7.3	8.3	0.070	7.6	7.6	0.974
50–64	29.2	28.7		29.1	28.4		17.2	17.6		17.2	17.0	
65–79	38.7	37.2		38.3	38.6		37.8	35.7		37.3	37.3	
80+	21.9	23.3		22.2	22.2		37.8	38.4		37.9	38.1	
Charlson score												
0–1	43.5	43.6	0.214	43.6	43.5	0.996	28.1	28.7	0.796	28.3	28.4	0.796
2–3	29.8	32.0		30.3	30.4		36.8	36.4		36.8	37.1	
4+	26.7	24.4		26.2	26.2		35.0	34.8		35.0	34.6	
Medical history												
Hypertension												
Yes	76.8	75.5	0.375	76.5	77.0	0.529	78.7	76.7	0.017	78.2	78.1	0.850
No	23.2	24.5		23.5	23.0		21.3	23.3		21.8	21.9	
Diabetes mellitus												
Yes	48.6	48.9	0.860	48.7	48.7	0.980	45.8	44.9	0.360	45.6	45.4	0.781
No	51.4	51.1		51.3	51.3		54.2	55.1		54.4	54.6	
Myocardial infarction												
Yes	12.7	13.8	0.343	13.0	12.5	0.459	9.3	10.4	0.079	9.6	9.6	0.961
No	87.3	86.2		87.0	87.5		90.7	89.6		90.4	90.4	
Heart Failure												
Yes	15.6	19.5	0.002	16.5	16.4	0.917	43.0	45.6	0.009	43.7	43.5	0.828
No	84.4	80.5		83.5	83.6		57.0	54.4		56.3	56.5	
Atrial fibrillation or flutter												
Yes	9.7	11.9	0.028	10.2	10.0	0.788	28.8	28.1	0.415	28.7	28.9	0.642
No	90.3	88.1		89.8	90.0		71.2	71.9		71.3	71.1	
Peripheral vascular disease												
Yes	3.7	3.5	0.801	3.6	3.7	0.865	4.1	4.2	0.749	4.2	4.1	0.835
No	96.3	96.5		96.4	96.3		95.9	95.8		95.9	95.9	
Renal disease												
Yes	18.6	17.9	0.600	18.5	18.6	0.882	19.8	19.9	0.920	19.8	19.5	0.552
No	81.4	82.1		81.5	81.4		80.2	80.1		80.2	80.5	
In-hospital treatment												
PCI												
Yes	59.1	53.3	<0.001	57.7	57.4	0.748	3.7	2.3	<0.001	3.4	3.3	0.583
No	40.9	46.7		42.3	42.6		96.3	97.7		96.6	96.8	
Intensive care unit use												
Yes	86.0	86.5	0.670	86.1	86.1	0.934	21.7	22.4	0.398	21.9	21.8	0.966
No	14.0	13.5		13.9	13.9		78.3	77.6		78.1	78.2	
Surgery use												
Yes	9.5	9.2	0.752	9.4	10.0	0.384	4.2	4.6	0.253	4.3	4.2	0.850
No	90.5	90.8		90.6	90.0		95.8	95.4		95.7	95.8	
Length of stay, d												
<5	41.4	38.3	0.043	40.7	40.7	0.993	42.6	39.4	<0.001	41.8	41.8	0.916
6–9	30.3	29.8		30.2	30.3		32.5	31.2		32.2	32.4	
10+	28.2	31.9		29.1	29.0		24.9	29.4		26.1	25.9	
Baseline medications												
Aspirin												
Yes	94.4	92.9	0.055	94.1	94.2	0.773	48.5	48.1	0.683	48.4	48.3	0.832
No	5.6	7.1		5.9	5.8		51.5	51.9		51.6	51.7	
β-Blocker												
Yes	63.8	62.7	0.499	63.6	64.0	0.707	38.7	39.1	0.693	38.8	38.6	0.739
No	36.2	37.3		36.4	36.0		61.3	60.9		61.2	61.4	
Statin												
Yes	58.3	61.7	0.041	59.1	59.4	0.800	16.8	16.7	0.878	16.8	16.8	0.995
No	41.7	38.3		40.9	40.6		83.2	83.3		83.2	83.2	
ACEI/ARB												
Yes	71.6	73.8	0.141	72.1	71.6	0.644	58.9	58.9	0.961	58.9	58.6	0.681
No	28.4	26.2		28.0	28.4		41.1	41.1		41.1	41.4	
Low income												
Yes	1.9	2.1	0.779	2.0	2.1	0.669	2.1	2.6	0.075	2.2	2.2	0.679
No	98.1	97.9		98.0	97.9		97.9	97.4		97.8	97.8	
Rural residence												
Yes	24.4	23.0	0.317	24.1	23.9	0.872	27.4	26.3	0.184	27.2	27.3	0.776
No	75.6	77.0		76.0	76.1		72.6	73.7		72.8	72.7	
Number of hospitalizations during the past year												
0	66.9	63.8	0.105	66.1	65.6	0.784	52.1	48.1	<0.001	51.1	51.5	0.798
1	18.2	20.7		18.9	19.4		24.5	25.0		24.6	24.5	
2+	14.9	15.5		15.1	15.0		23.4	26.9		24.3	24.0	
Number of outpatient visits during the past year												
Low	32.7	37.6	<0.001	33.8	33.4	0.889	31.9	40.0	<0.001	33.9	33.9	0.982
Medium	32.0	35.9		33.0	33.4		32.3	34.0		32.8	32.9	
High	35.3	26.5		33.2	33.2		35.7	26.0		33.3	33.3	
Hospital Characteristics												
Hospital volume												
Low	35.3	28.2	<0.001	33.7	34.4	0.783	35.5	31.4	<0.001	34.4	34.3	0.819
Medium	33.9	33.5		33.8	33.6		33.9	33.2		33.7	34.1	
High	30.8	38.3		32.5	32.1		30.7	35.4		31.9	31.6	
Hospital level												
Academic medical center	53.9	62.4	<0.001	55.8	55.0	0.742	31.5	40.1	<0.001	33.7	33.7	0.967
Regional	43.2	35.7		41.6	42.3		53.0	46.7		51.4	51.3	
District	2.9	1.9		2.7	2.7		15.5	13.2		14.9	15.0	
Teaching												
Yes	98.5	98.9	0.377	1.4	1.4	0.874	88.1	89.4	0.043	88.4	88.4	0.946
No	1.5	1.1		98.6	98.6		11.9	10.6		11.6	11.6	
Location												
Taipei	34.0	37.4	<0.001	34.7	33.8	0.934	32.9	37.0	<0.001	33.9	33.9	0.997
Northern	10.9	13.9		11.6	11.6		13.7	13.0		13.5	13.4	
Central	15.2	18.3		16.0	16.2		18.6	18.7		18.7	18.8	
Southern	18.2	13.5		17.2	17.8		16.0	12.6		15.1	15.2	
Kao-Ping	18.4	12.9		17.1	17.3		15.2	15.6		15.3	15.3	
Eastern	3.3	4.0		3.4	3.3		3.6	3.1		3.4	3.4	

NSTEMI indicates non-ST-segment-elevation myocardial infarction; and PCI, percutaneous coronary intervention.

^a^Based on propensity score weighting.

### Associations between Early Follow-Up and 30-Day Readmission

Table 3 shows the unadjusted associations of early physician follow-up, and patient and hospital characteristics with 30-day readmission. For each condition, early follow-up with a physician, early follow-up with the same physician, and early follow-up with a cardiologist were associated with reduced 30-day readmission. For two conditions, patient and hospital characteristics associated with 30-day readmission were patient sex, age, comorbid conditions, medical history, in-hospital treatment, length of stay, baseline medications, medications within 7 days of discharge, low income, number of hospitalizations during the past year, number of outpatient visits during the past year, hospital volume, level, and teaching status.

**Table 3:** 30-day all-cause readmission rates by follow-up and baseline characteristics.

	NSTEMI		Heart failure	
	%	P value	%	P value
Early follow-up				
Yes	17.4	<0.001	20.7	<0.001
No	28.3		31.2	
Early follow-up with the same physician				
Yes	14.4	<0.001	18.1	<0.001
No	27.0		29.2	
Early follow-up with a cardiologist				
Yes	15.3	<0.001	18.1	<0.001
No	23.6		26.5	
Patient Characteristics				
Sex				
Male	18.5	<0.001	23.8	0.205
Female	22.9		22.9	
Age (yr)				
18–49	11.7	<0.001	18.4	<0.001
50–64	16.9		20.0	
65–79	21.2		23.3	
80+	25.5		25.9	
Charlson score				
0–1	12.3	<0.001	17.9	<0.001
2–3	22.0		22.0	
4+	30.2		29.1	
Medical history				
Hypertension				
Yes	21.1	<0.001	23.7	0.086
No	16.0		22.2	
Diabetes mellitus				
Yes	24.3	<0.001	24.8	<0.001
No	15.8		22.1	
Myocardial infarction				
Yes	22.9	0.040	26.5	0.005
No	19.5		23.0	
Heart Failure				
Yes	30.2	<0.001	25.6	<0.001
No	17.9		21.6	
Atrial fibrillation or flutter				
Yes	24.7	0.005	22.4	0.090
No	19.4		23.7	
Peripheral vascular disease				
Yes	25.3	0.066	28.9	0.001
No	19.7		23.1	
Renal disease				
Yes	30.3	<0.001	28.6	<0.001
No	17.6		22.0	
In-hospital treatment				
PCI				
Yes	13.4	<0.001	17.7	0.004
No	28.8		23.5	
Intensive care unit use				
Yes	19.9	0.751	26.3	<0.001
No	20.4		22.5	
Surgery use				
Yes	27.4	<0.001	26.5	0.069
No	19.2		23.2	
Length of stay (d)				
<5	14.4	<0.001	19.7	<0.001
6–9	18.8		22.7	
10+	28.9		29.9	
Baseline medications				
Aspirin				
Yes	19.5	0.005	22.5	0.028
No	26.2		24.1	
β-Blocker				
Yes	19.2	0.074	21.5	<0.001
No	21.3		24.5	
Statin				
Yes	17.8	<0.001	21.5	0.020
No	23.0		23.7	
ACEI/ARB				
Yes	18.9	0.003	21.8	<0.001
No	22.6		25.6	
Medications within 7 days of discharge				
Aspirin				
Yes	14.4	<0.001	19.4	<0.001
No	25.8		24.8	
β-Blocker				
Yes	15.1	<0.001	18.1	<0.001
No	22.7		25.0	
Statin				
Yes	13.8	<0.001	17.0	<0.001
No	23.0		24.0	
ACEI/ARB				
Yes	15.1	<0.001	17.1	<0.001
No	22.6		26.2	
Medications within 14 days of discharge				
Aspirin				
Yes	14.6	<0.001	19.3	<0.001
No	31.5		25.4	
β-Blocker				
Yes	15.3	<0.001	18.1	<0.001
No	24.4		25.8	
Statin				
Yes	13.8	<0.001	17.6	<0.001
No	25.0		24.2	
ACEI/ARB				
Yes	14.6	<0.001	17.0	<0.001
No	24.9		27.8	
Low income				
Yes	32.7	0.001	27.9	0.059
No	19.7		23.2	
Rural residence				
Yes	21.2	0.219	23.4	0.876
No	19.5		23.3	
Number of hospitalizations during the past year				
0	15.5	<0.001	18.2	<0.001
1	26.0		23.4	
2+	32.0		34.0	
Number of outpatient visits during the past year				
Low	14.8	<0.001	20.3	<0.001
Medium	19.7		23.3	
High	25.4		26.5	
Hospital Characteristics				
Hospital volume				
Low	21.2	0.041	25.5	<0.001
Medium	20.6		23.4	
High	17.9		21.0	
Hospital level				
Academic medical center	18.0	<0.001	20.0	<0.001
Regional	22.0		24.3	
District	28.4		27.6	
Teaching				
Yes	19.7	0.001	22.9	<0.001
No	36.2		26.9	
Location				
Taipei	18.1	0.127	22.1	0.134
Northern	19.1		23.1	
Central	20.3		24.1	
Southern	22.1		25.1	
Kao-Ping	21.1		23.7	
Eastern	23.3		23.6	

NSTEMI indicates non-ST-segment-elevation myocardial infarction; and PCI, percutaneous coronary intervention.

The results of the series of Cox proportional hazard models with robust sandwich variance estimates and propensity score weighting as provided in Table 4 demonstrate the associations between early follow-up and 30-day readmission, after adjustment for patient and hospital characteristics. In model 1, early physician follow-up was associated with a lower hazard ratio of all-cause readmission compared with no early physician follow-up for patients with NSTEMI (hazard ratio [HR], 0.47; 95% confidence interval [CI], 0.39–0.57), and for patients with heart failure (HR, 0.54; 95% CI, 0.48–0.60). Early physician follow-up was also associated with lower 30-day cardiovascular-cause and same-cause readmission.

**Table 4:** Adjusted relationships between early physician follow-up and 30-day readmission.

	All-cause		Cardiovascular-cause		Same-cause	
	HR	(95% CI)	HR	(95% CI)	HR	(95% CI)
NSTEMI						
Model 1: early physician follow-up^a^	N = 5008					
Yes (ref: No)	0.47	(0.39–0.57)	0.48	(0.40–0.57)	0.59	(0.41–0.86)
Model 2: early physician follow-up^b^	N = 3841					
Same physician follow-up (ref: No)	0.56	(0.48–0.65)	0.56	(0.47–0.66)	0.62	(0.38–0.99)
Cardiologist follow-up (ref: No)	0.97	(0.81–1.16)	1.03	(0.86–1.24)	0.81	(0.54–1.23)
Model 3: 14-Day physician follow-up^a^	N = 5008					
Yes (ref: No)	0.18	(0.14–0.24)	0.20	(0.15–0.26)	0.26	(0.17–0.39)
Model 4: 14-Day physician follow-up^b^	N = 4664					
Same physician follow-up (ref: No)	0.53	(0.44–0.62)	0.51	(0.43–0.61)	0.59	(0.39–0.92)
Cardiologist follow-up (ref: No)	0.85	(0.73–1.00)	0.89	(0.76–1.05)	0.85	(0.58–1.24)
Heart failure						
Model 1: early physician follow-up^a^	N = 13577					
Yes (ref: No)	0.54	(0.48–0.60)	0.57	(0.51–0.64)	0.59	(0.52–0.66)
Model 2: early physician follow-up^b^	N = 10164					
Same physician follow-up (ref: No)	0.69	(0.62–0.76)	0.69	(0.62–0.76)	0.77	(0.68–0.87)
Cardiologist follow-up (ref: No)	0.94	(0.85–1.04)	0.99	(0.90–1.10)	1.03	(0.91–1.17)
Model 3: 14-Day physician follow-up^a^	N = 13577					
Yes (ref: No)	0.28	(0.25–0.32)	0.31	(0.27–0.34)	0.32	(0.28–0.36)
Model 4: 14-Day physician follow-up^b^	N = 12197					
Same physician follow-up (ref: No)	0.63	(0.58–0.68)	0.64	(0.59–0.70)	0.71	(0.64–0.80)
Cardiologist follow-up (ref: No)	0.94	(0.86–1.03)	1.00	(0.91–1.09)	1.05	(0.94–1.18)

CI indicates confidence interval; HR, hazard ratio; NSTEMI, non-ST-segment-elevation myocardial infarction; and PCI, percutaneous coronary intervention.

^a^Models included sex, age, comorbid conditions, medical history, in-hospital treatment, length of stay, baseline medications, low income, rural residence, number of hospitalizations during the past year, number of office visits during the past year, hospital volume, level, teaching status, and location, and were weighted by the inverse of a propensity score

^b^Models included sex, age, comorbid conditions, medical history, in-hospital treatment, length of stay, baseline medications, medications within 7(14) days of discharge, low income, rural residence, number of hospitalizations during the past year, number of office visits during the past year, hospital volume, level, teaching status, and location.

To further determine the individual and combined associations of same physician and cardiologist follow-up with 30-day readmission among patients with early follow-up, we did not find the synergistic associations between the effects of same physician and cardiologist follow-up. Therefore, an interaction term between same physician and cardiologist follow-up was removed in model 2. Same physician follow-up was associated with a lower hazard ratio of all-cause readmission compared with different physician follow-up for patients with NSTEMI (HR, 0.56; 95% CI, 0.48–0.65), and for patients with heart failure (HR, 0.69; 95% CI, 0.62–0.76). Same physician follow-up was also associated with lower 30-day cardiovascular-cause and same-cause readmission. The robustness of our primary results were evaluated by sensitivity analysis. Results were similar when we changed the transitional period from 7 days to 14 days.

## Discussion

This study was the first research using nationwide population-based data to examine the association between early physician follow-up and 30-day readmission, and the relative association of early same physician and early cardiologist follow-up with 30-day readmission for NSTEMI and heart failure. For each condition, we found that early physician follow-up was associated with decreased 30-day readmission compared with no early physician follow-up. Moreover, early follow-up with the same physician was associated with lower 30-day readmission compared with early follow-up with a different physician.

In Taiwan, about 75% percent of hospitalized patients with NSTEMI and heart failure had an outpatient visit within 7 days of discharge. Most early follow-up care was handled by the same physician who treated the patient during the index hospitalization, and usually by cardiologists. The rates of early physician, early same physician and early cardiologist follow-up in Taiwan were higher than those in the United States (38.3%, 18.1%, and 7.5%, respectively, for heart failure).[3] These results are because all hospitals in Taiwan are closed systems and are reimbursed for both inpatient and outpatient services on a fee-for-service basis by the National Health Insurance Administration (NHIA). Thus, physicians only employed by hospitals can be allowed to treat inpatients, and hospitals also use variable pay to encourage staff physicians to provide inpatient and outpatient services. Moreover, clinical guidelines recommend early physician follow-up. Therefore, most hospital physicians not only prescribe a week's supply of medicine at discharge, but also schedule a follow-up appointment within 7 days of discharge.

This study had two major findings about the association between early physician follow-up and 30-day readmission for NSTEMI and heart failure. First, we found that early follow-up with any physician was associated with lower 30-day readmission for each condition. This result is consistent with the finding of Hernandez et al.[3] Hernandez et al verified the association between hospital-level early physician follow-up and 30-day readmission among Medicare beneficiaries hospitalized for heart failure. However, the aggregate group-level (hospital-level) variable may be tapping into a different construct than its individual-level (patient-level) namesake.[41–43] Thus, the limitations of using an aggregate measure as a proxy for its individual-level namesake relate not only to measurement errors but also to construct validity, that is, whether it is indeed the same construct that is measured by both variables.[41, 43, 44]

It also must be noted that at the patient level, the association between time from hospital discharge to outpatient follow-up with a physician and risk of readmission is confounded by severity of illness. Patients who have more severe acute myocardial infarction or heart failure, or who are medically less stable are not only commonly seen sooner after hospital discharge but also at a greater risk of readmission.[3, 4] Therefore, we used a propensity score approach to correct for the selection bias. The use of a propensity score method can help reduce the selection bias and provide more valid analysis results. This study may support current clinical guidelines advocating for patients with heart failure or NSTEMI who have been discharged from hospital to receive early or timely outpatient follow-up with a physician for further assessment or treatment.[5, 7]

Another notable finding was that early same physician follow-up (physician continuity) was associated with a further reduction in 30-day readmission. This finding is consistent with that of van Walraven et al[23] and of McAlister et al.[19] In Ontario, Canada, Walraven et al found that 30-day follow-up with the same physician, rather than with another physician, was associated with reduced 30-day post-discharge mortality or non-elective readmission for non-elective medical or surgical conditions. In Alberta, Canada, McAlister et al found that 30-day follow-up with a familiar physician was associated with lower 3-month post-discharge mortality or urgent readmission for heart failure compared with no follow-up. Although at discharge disease progression is improved, remission is not achieved. Because of early follow-up with a different physician, if the physician does not know a particular patient’s disease progression, their progression could possibly be interpreted as a deterioration requiring readmission.[23] Moreover, early same physician follow-up is more likely to early determine therapeutic effectiveness and early identify complications of hospital therapies or procedures due to familiarity with the hospital course. Early evaluation and treatment could avoid more serious subsequent problems leading to readmission.

Some limitations existed in this study. First of all, patients were not assigned randomly. This is an observational and retrospective study; therefore, we cannot assign study subjects randomly. The propensity score weighting was adopted for alleviating selection bias.[25, 37–40] Moreover, although, like other studies using administrative databases, no information on disease severity was available for risk adjustment, we adjusted for patient age, comorbid conditions, intensive care unit use, and surgical operation, which are also important for the adjustment of disease complexity.[9, 19, 23–26] However, we cannot completely exclude the possibility of unmeasured confounders such as behavioral risk factors. Differences in outcomes between follow-up and non-follow-up groups may be confounded by unmeasured behavioral risk factors that may influence access to care. Second, this study defined early follow-up as an outpatient visit that occurred within 7 days of discharge. The 7-day window has previously been discussed and was chosen according to historical precedent and clinical plausibility.[3, 4] However, we also did a sensitivity analysis examining the association between 14-day follow-up and 30-day readmission.

Finally, in Taiwan, follow-up with a trained and specialized nurse (care manager) is not common until now. This may be because the service is not reimbursed by the NHIA. Based on two related studies, one study regarding discharged patient with heart failure in Northern California, United States finds that early outpatient follow-up with a physician is associated with a lower chance of 30-day readmission, but telephone follow-up with a trained and specialized nurse or pharmacist is not.[45] Another study regarding ambulatory patients with heart failure in the Apulia Region of Italy finds that the introduction of care managers into the primary health care system has the potential of reducing readmissions because of the strong partnership between the care manager and the patient and the collaboration between the physician and the care manager.[46] Future research needs to examine whether the effect of early follow-up visit with a care manager is the same as that with a physician among discharged patients with heart failure or acute myocardial infarction.

## Conclusion

Our national population-based study showed that 7-day physician follow-up was associated with lower 30-day readmission, and physician continuity (7-day same physician follow-up) was associated with much lower 30-day readmission for patients with NSTEMI and those with heart failure. This study may provide evidence in support of guidelines recommending scheduling an early follow-up visit after discharge, and it may provide an evidenced-based approach to improve 30-day readmission following NSTEMI and heart failure.
